# Distribution of *Legionella* and bacterial community composition among regionally diverse US cooling towers

**DOI:** 10.1371/journal.pone.0189937

**Published:** 2017-12-20

**Authors:** Anna C. Llewellyn, Claressa E. Lucas, Sarah E. Roberts, Ellen W. Brown, Bina S. Nayak, Brian H. Raphael, Jonas M. Winchell

**Affiliations:** 1 Laboratory Leadership Service, Centers for Disease Control and Prevention, Atlanta, GA, United States of America; 2 Pneumonia Response and Surveillance Laboratory, Respiratory Disease Branch, Centers for Disease Control and Prevention, Atlanta, GA, United States of America; 3 Water Quality Division, Pinellas County Utilities, Largo, FL, United States of America; Defense Threat Reduction Agency, UNITED STATES

## Abstract

Cooling towers (CTs) are a leading source of outbreaks of Legionnaires’ disease (LD), a severe form of pneumonia caused by inhalation of aerosols containing *Legionella* bacteria. Accordingly, proper maintenance of CTs is vital for the prevention of LD. The aim of this study was to determine the distribution of *Legionella* in a subset of regionally diverse US CTs and characterize the associated microbial communities. Between July and September of 2016, we obtained aliquots from water samples collected for routine *Legionella* testing from 196 CTs located in eight of the nine continental US climate regions. After screening for *Legionella* by PCR, positive samples were cultured and the resulting *Legionella* isolates were further characterized. Overall, 84% (164) were PCR-positive, including samples from every region studied. Of the PCR-positive samples, *Legionella* spp were isolated from 47% (78), *L*. *pneumophila* was isolated from 32% (53), and *L*. *pneumophila* serogroup 1 (Lp1) was isolated from 24% (40). Overall, 144 unique *Legionella* isolates were identified; 53% (76) of these were *Legionella pneumophila*. Of the 76 *L*. *pneumophila* isolates, 51% (39) were Lp1. *Legionella* were isolated from CTs in seven of the eight US regions examined. 16S rRNA amplicon sequencing was used to compare the bacterial communities of CT waters with and without detectable *Legionella* as well as the microbiomes of waters from different climate regions. Interestingly, the microbial communities were homogenous across climate regions. When a subset of seven CTs sampled in April and July were compared, there was no association with changes in corresponding CT microbiomes over time in the samples that became culture-positive for *Legionella*. *Legionella* species and Lp1 were detected frequently among the samples examined in this first large-scale study of *Legionella* in US CTs. Our findings highlight that, under the right conditions, there is the potential for CT-related LD outbreaks to occur throughout the US.

## Introduction

Legionellae are Gram-negative opportunistic bacterial pathogens common to soil and freshwater environments. These bacteria are the causative agents of Legionnaires’ disease (LD), a severe form of pneumonia that primarily affects adults who are 50 years or older, have a history of smoking or chronic lung disease, or are immunocompromised. *Legionella* infections are primarily spread via inhalation of contaminated aerosols from man-made water systems and devices such as showers, whirlpool spas, and cooling towers (CTs) [1]. *Legionella* is the leading cause of deaths from waterborne outbreaks in the US [2] and the rate of reported cases of legionellosis in the US increased nearly 4-fold from 2000 to 2014 [3, 4], highlighting the urgency of this public health threat.

While approximately half of the ~60 identified *Legionella* species (Lspp) have been shown to cause disease [5, 6], up to 92% of reported cases of legionellosis in the US are caused by *Legionella pneumophila* serogroup 1 (Lp1) [1]. The high incidence of reported cases caused by Lp1 may be influenced in part by the fact that Lp1 is the target for the urinary antigen test, the most widely used LD diagnostic [1]. *L*. *pneumophila* serogroup 6, *L*. *longbeachae*, and *L*. *micdadei* are among the next most commonly detected agents of legionellosis [1].

CTs are a part of the air-conditioning systems often present in large buildings, such as hotels or hospitals, which use water to efficiently cool air via heat transfer. Environmental microbes can flourish in CT systems that are not properly maintained. The presence of sediment, nutrients, heterotrophic biofilm, and amoebae in warm water combined with insufficient biocide treatment can result in high numbers of legionellae [7, 8]. These microbes can then become aerosolized in the spray or mist generated by the tower. In some outbreaks, cooling tower plumes have been reported to disperse over several kilometers [9]. Susceptible individuals who inhale *Legionella*-containing aerosols are at risk for developing LD. CTs have been linked to many reported LD outbreaks [10–12].

Technologies such as high-throughput 16S rRNA amplicon sequencing have facilitated research into the microbiomes of the natural and built environments [13]. However, reports on the microbiomes of water in these systems are limited and primarily related to potable water [14, 15]. Initial studies have concentrated on the microbiomes of CTs from a specific site or region [16–18]. A two-year study of the microbiome of a single cooling tower in Germany revealed a diverse bacterial community with *Legionella* abundances ranging between 0.06%-6.0% [18]. However, a large survey of CT microbiomes has not been reported to date.

Currently there are no published studies that identify the presence of *Legionella* in US CTs with broad geographic range. Additionally, relationships between *Legionella* and other components of bacterial communities present in CTs have not been extensively studied. To better understand the distribution of *Legionella* and the bacterial community composition in US CTs with no known association with disease, we assayed CT water samples taken for routine *Legionella* testing from 196 sites across the US using multiplex PCR, *Legionella* culture, and 16S rRNA amplicon sequencing.

## Materials & methods

### Water sample collection

A subset of water samples submitted to four commercial water testing companies for routine testing were shared with CDC. The sample collection protocols of the testing laboratories require addition of sodium thiosulfate to neutralize any residual disinfectant. Bulk water samples (25–50 ml) were collected from the CT basin. We examined a total of 196 samples collected from separate towers located in each of the continental US climate regions as defined by the National Oceanic and Atmospheric Administration (NOAA) with the exception of the West North Central region. Additionally, in April and July of 2016, samples from seven CTs were collected by a local government utility water quality laboratory in Florida in parallel with routine water sampling specifically for this study. Samples were typically batch shipped to the CDC at ambient temperature in 1–2 week intervals from the initial testing laboratories.

### DNA extraction

To prepare water samples for DNA extraction, 15mL were centrifuged at 4700 x g at ambient temperature for 20 minutes. Supernatant was removed and the pellet resuspended in 500 μl sterile nuclease-free water (Promega, Madison, WI) and 2 μl of Ready-Lyse Lysozyme Solution (Epicentre, Madison, WI) were added. Next, samples were incubated at 37°C for 30 minutes with shaking. DNA extraction was then performed on 400 μl of prepared sample using the Roche MagNApure Compact instrument (Roche Applied Science, Indianapolis, IN) and Roche MagNApure Compact Nucleic Acid Isolation Kit I reagents (Roche). *Legionella* isolate gDNA was also extracted using the Roche MagNApure Compact system.

### *Legionella* multiplex real-time PCR

Detection of *Legionella* DNA was performed using a previously reported real-time PCR assay [19, 20]. An updated PCR protocol and internal inhibition control were generously shared by Dr. Kimberlee Musser (Wadsworth Center, New York State Department of Health). The assay targets three distinct DNA regions and gives three independent results: 1) presence of a 23S rRNA gene region which is common to all *Legionella* species (Lspp) and serogroups, 2) presence of a conserved portion of the *mip* gene common to all *L*. *pneumophila* (Lp) serogroups, and 3) presence of the *wzm* gene specific for *L*. *pneumophila* serogroup 1 (Lp1). Briefly, 5 μL of nucleic acid extract from a water sample or *Legionella* isolate were added to a PCR master mix containing 0.2 μL of each primer (50 μM), 0.25 μL of each probe (25 μM), 12.5 μL Quanta PerfeCTa Multiplex qPCR SuperMix (Quanta Biosciences, Gaithersburg, MD), 4.8 μL of nuclease-free water (Promega), and 0.5 μL internal inhibition control (optimized to be detected at a crossing threshold value of 30–35). The reactions were performed in 96-well optical reaction plate in an Applied Biosystems 7500 Fast Real-Time PCR instrument (ThermoFisher, Waltham, MA) under the following cycling conditions: 3 min at 95°C and then 40 cycles of 15 seconds at 95°C followed by 45 seconds at 60°C. Using this assay, a DNA extract containing Lp1 DNA would result in positive crossing threshold values for all three targets while a sample containing only *Legionella* DNA from species other than *L*. *pneumophila* would result in positive crossing threshold values for the *23S* target only. An Lp1 gDNA positive control (optimized to be detected at a crossing threshold value of 30–35) and nuclease-free water (Promega) negative control were run on each 96-well plate. All samples and controls were run at least in duplicate.

### *Legionella* culture

Water samples that tested positive for *Legionella* by PCR were processed and plated for culture as previously described [21]. Briefly, direct culture was performed by plating 100 μl of CT water directly onto BCYE agar plates with and without antibiotic selection [polymyxin B (1000 U/L), vancomycin (5mg/mL), cyclohexamide (80 mg/mL), glycine (2 g/L)]. Additionally, a 10 mL aliquot of each sample was centrifuged for 20 minutes at 4700 x g at ambient temperature, the pellet resuspended in 500 μl sterile water, acid-treated (0.2M KCL, 0.2M HCL) for 15 minutes, and then 100 μl plated onto BCYE with antibiotic selection and incubated at 35°C with 2% CO_2_. At 3 and 7 days post-inoculation, a dissecting microscope was used to select suspect *Legionella* colonies and the colonies streaked onto BCYE agar with and without cysteine. Cysteine auxotrophs were then streaked for isolation on BCYE agar. The accuracy of *Legionella* enumeration can be variable among testing laboratories [22] and was not within the intended scope of this study.

### *Legionella* isolate characterization

DNA from *Legionella* isolates identified by cysteine auxotrophy was analyzed by multiplex PCR as described above. Isolates identified by PCR as Lp1 were frozen for future reference. Isolates identified by PCR as Lp but not serogroup 1 were further characterized by slide agglutination and direct fluorescence antibody testing as previously described [23, 24]. Species other than Lp were identified by *mip* sequencing using a published method [25]. Sanger sequence reads of isolate *mip* sequences were assembled into final contigs which were used to query the National Center for Biotechnology Information (NCBI) GenBank nucleotide database using the Basic Local Alignment Search Tool (BLAST). Alignments of 96% or greater identity over at least 98% of double stranded consensus were considered a species match. In cases where *mip* sequencing could not resolve between highly related species, isolates were further assayed by slide agglutination using species-specific antibodies. Isolates with low or poor alignment to known *Legionella* species or that would not amplify *mip* with the traditional primers were categorized as potential novel *Legionella* species.

### 16S rRNA amplicon sequencing

Amplicon libraries of the bacterial *16S* rRNA gene from water samples and control mock communities were prepared according to the Illumina MiSeq “16S Metagenomic Sequencing Library Preparation” protocol (Illumina, San Diego, CA) and sequenced by an Illumina MiSeq instrument using a MiSeq v3 Reagent Kit according to the “Illumina 16S Metagenomic Sequencing” protocol (Illumina). Sequence data were deposited in the NCBI Sequence Read Archive under BioProject ID: PRJNA391126. Raw sequence data from the forward read were cleaned to remove reads with primer mismatches, missing or low (below 25) quality scores, less than 150 base pairs or greater than 350 base pairs, and more than 6 ambiguous bases. Data from samples with fewer than 100,000 remaining reads were discarded and the sample was resequenced. Sequence reads were further analyzed using Quantitative Insights into Microbial Ecology (QIIME) open source software [26] to determine the abundance of operational taxonomic units (OTUs) present in each sample. OTUs were defined as a subset of reads sharing ≥97% sequence identity and taxonomic identities were determined with the default classifier used in QIIME (i.e. Ribosomal Database Project).

### Data analysis

For PCR and culture results, a simple descriptive analysis was performed to examine relationships. The non-parametric Spearman correlation coefficient (assuming non-Gaussian distribution) r value was used to determine the significance of the abundance of *Legionella* DNA versus the number of taxa in the samples. Significance of the relationships between PCR and culture results and *Legionella* DNA abundance and total number of taxa present in a sample was determined via unpaired t test. The Mann-Whitney test was used to compare the relative abundance of Proteobacteria, *Comamonadaceae*, and *Pseudomonadaceae* in samples with different PCR and culture results.

## Results

### *Legionella* DNA is prevalent among US cooling tower water samples

**Fig 1:**
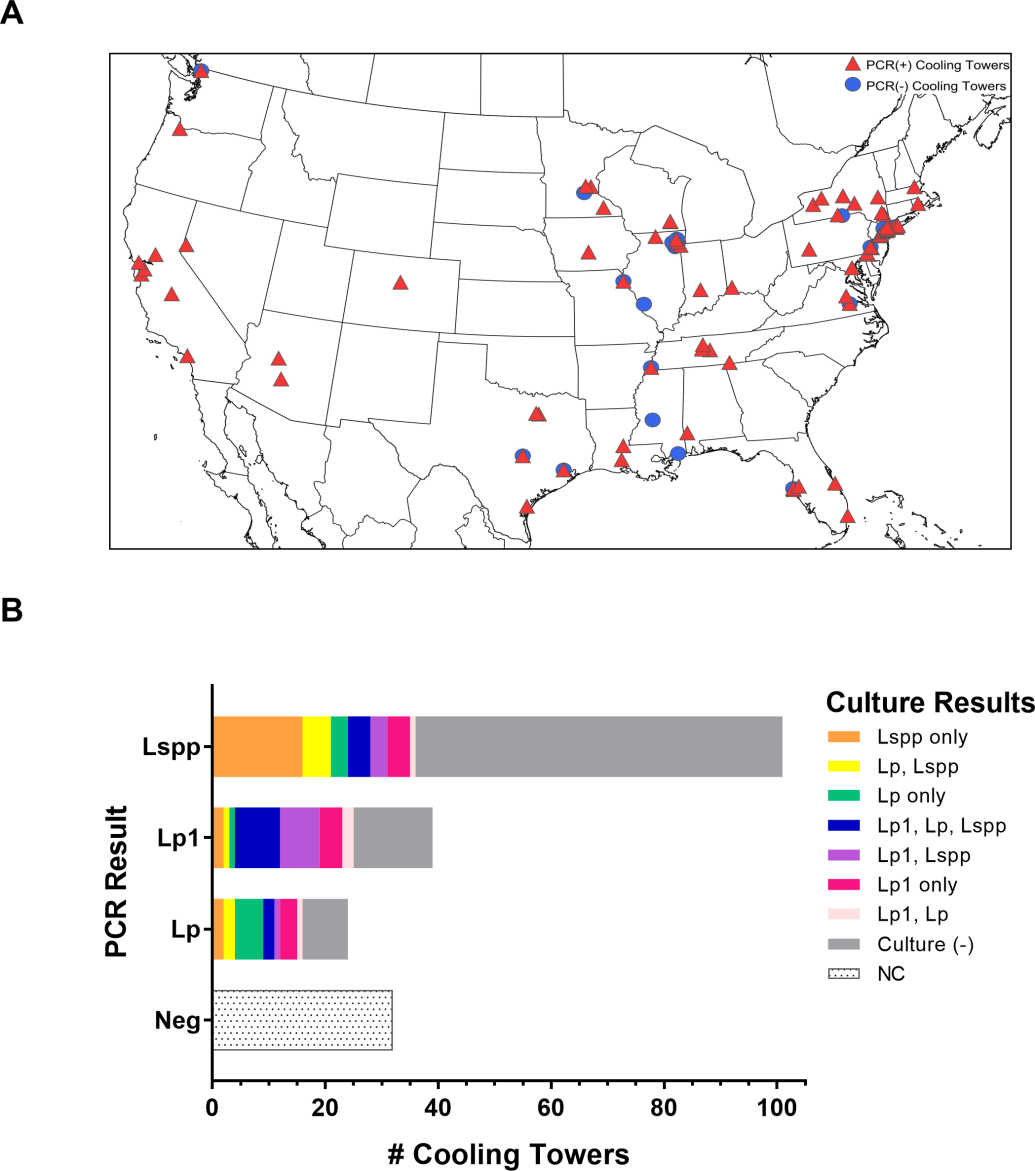
Cooling tower locations and PCR and culture results overview. The CT samples in this study were from geographic locations across the US (A). The approximate longitude and latitude of the city for each CT location is plotted on the map which was generated with SimpleMappr (www.simplemappr.net). CTs that were positive for *Legionella* DNA by PCR are represented by red triangles [PCR (+)] and those negative by PCR are shown as blue circles [PCR (-)] (A). PCR (+) refers to a positive result for any of the three discrete probes in the PCR assay (Lspp, Lp, Lp1) (A). Each sample was assayed by PCR (A, B) and PCR-positive samples underwent culture (B). Samples that yielded no isolates were culture-negative [Culture (-)]. PCR-negative samples were not cultured (NC).

A total of 196 CT water samples were received for testing during the summer of 2016. Initial screening of the CT samples using multiplex PCR indicated the presence of *Legionella* DNA (Lspp, Lp, or Lp1) in 164 (84%) samples (Fig 1A). Of the PCR-positive results, 39 (24%) were positive for Lp1, 24 (15%) for Lp but not Lp1, 101 (62%) for Lspp alone, and 2 were identified by the internal control to contain contaminants that resulted in PCR-inhibition (Fig 1B).

### Diverse *Legionella* isolated from PCR-positive cooling tower water samples

The 164 PCR-positive samples and the 2 PCR-inhibited samples were cultured and resulting *Legionella* isolates were characterized. Seventy-nine (47%) of these 166 samples, including one of the PCR-inhibited samples, were culture-positive, 41 (52%) of which were positive for more than one type of Lspp or Lp serogroup isolate (Fig 1B). Lp was recovered from 53 (32%) cultured samples. Lp1 was isolated from 40 samples, 19 (47%) of which were not specifically identified by PCR to contain the Lp1 DNA target (Fig 1B). In addition, at least one other Lp serogroup or Lspp isolate was recovered from 29 (72%) of the Lp1 culture-positive samples. (Fig 1B). Notably, the overall PCR crossing threshold values for detection of *Legionella* DNA did not correlate with culturability of *Legionella* bacteria (S1 Fig).

### *Legionella* detected in CT samples across US climate regions

**Fig 2:**
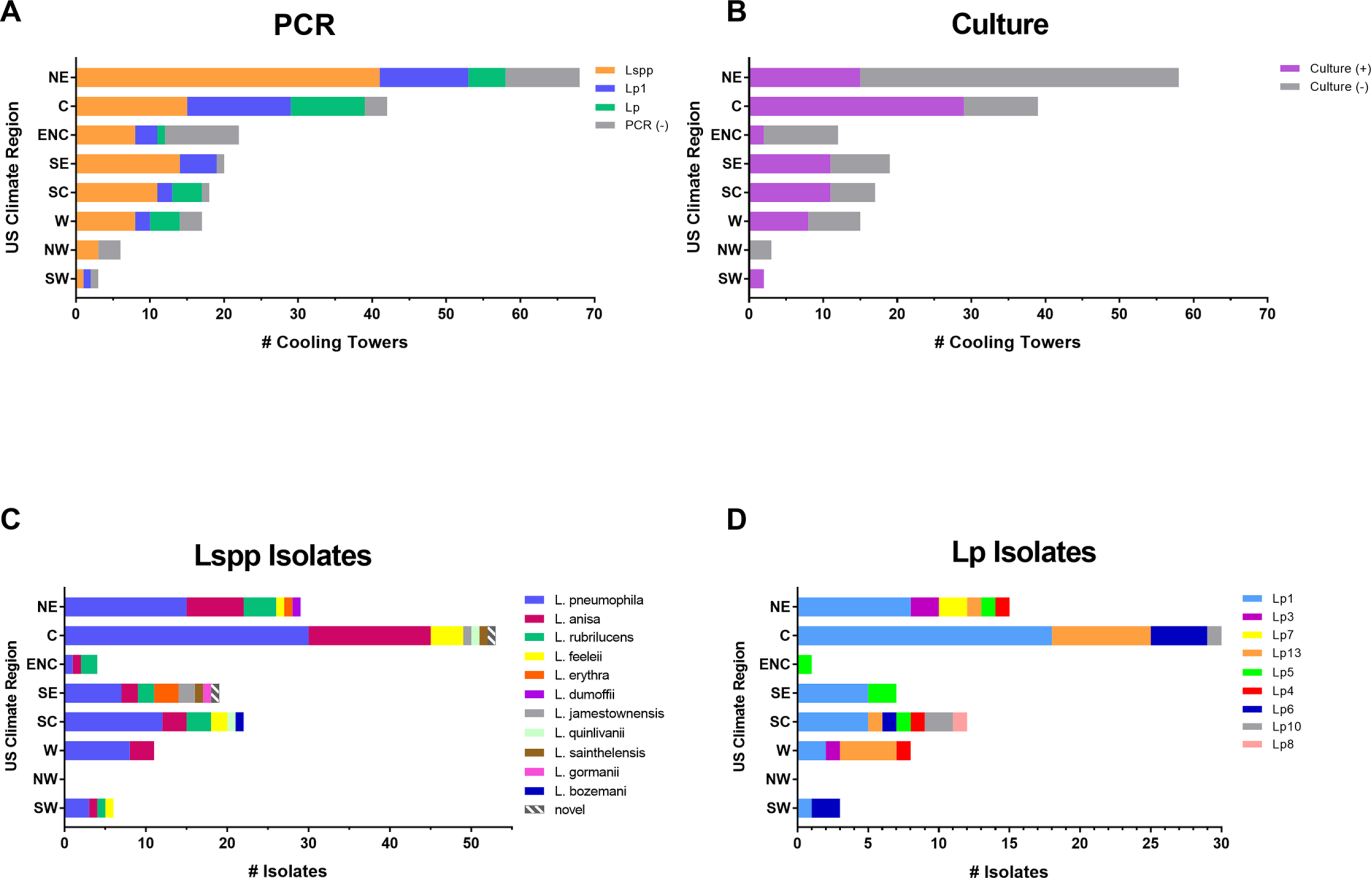
Geographic distribution of results. Samples from eight US climate regions [Central (C), East North Central (ENC), Northeast (NE), Northwest (NW), South [Central] (SC), Southeast (SE), Southwest (SW), and West (W)] were assayed by PCR (A) and culture (B). Specimens that were positive for any of the targets by PCR underwent culture. Isolates were identified to the species (C) and *L*. *pneumophila* serogroup (D) level.

The National Oceanic and Atmospheric Administration (NOAA) recognizes nine climate regions within the US: Central (C), East North Central (ENC), Northeast (NE), Northwest (NW), South [Central] (SC), Southeast (SE), Southwest (SW), West (W), and West North Central [27]. While we were unable to include CTs from the West North Central region in this study, *Legionella* DNA was detected in CTs from each of the eight regions contributing samples ranging from 50% (n = 6) in the NW region to 95% (n = 20) in the SE region (Fig 2A). While 47% of all PCR-positive samples (n = 164) were culture-positive, the regional percentages of culture-positive samples were more highly variable than PCR-positive results (Fig 2B). Excluding regions with <10 samples (NW and SW), positive culture results among PCR-positive samples ranged from 17% (n = 12) in the ENC region to 77% (n = 39) in the C region. A diverse range of Lspp were recovered across all regions (Fig 2C). Of all isolates recovered (n = 144), the most common species were *L*. *pneumophila* (53%), *L*. *anisa* (22%), and *L*. *rubrilucens* (9%) (Fig 2C). Though many different serogroups of *L*. *pneumophila* were recovered and identified by DFA, 51% (n = 76) of these were Lp1, which comprised the highest percentage of *L*. *pneumophila* isolates from nearly every region (Fig 2D). Importantly, though the majority of the PCR-positive results in each region identified Lspp only (Fig 2A), Lp and Lp1 isolates were often recovered from these same samples (Figs 1A, 2C and 2D).

### *Legionellaceae* relative abundance has a positive correlation with microbiome diversity

**Fig 3:**
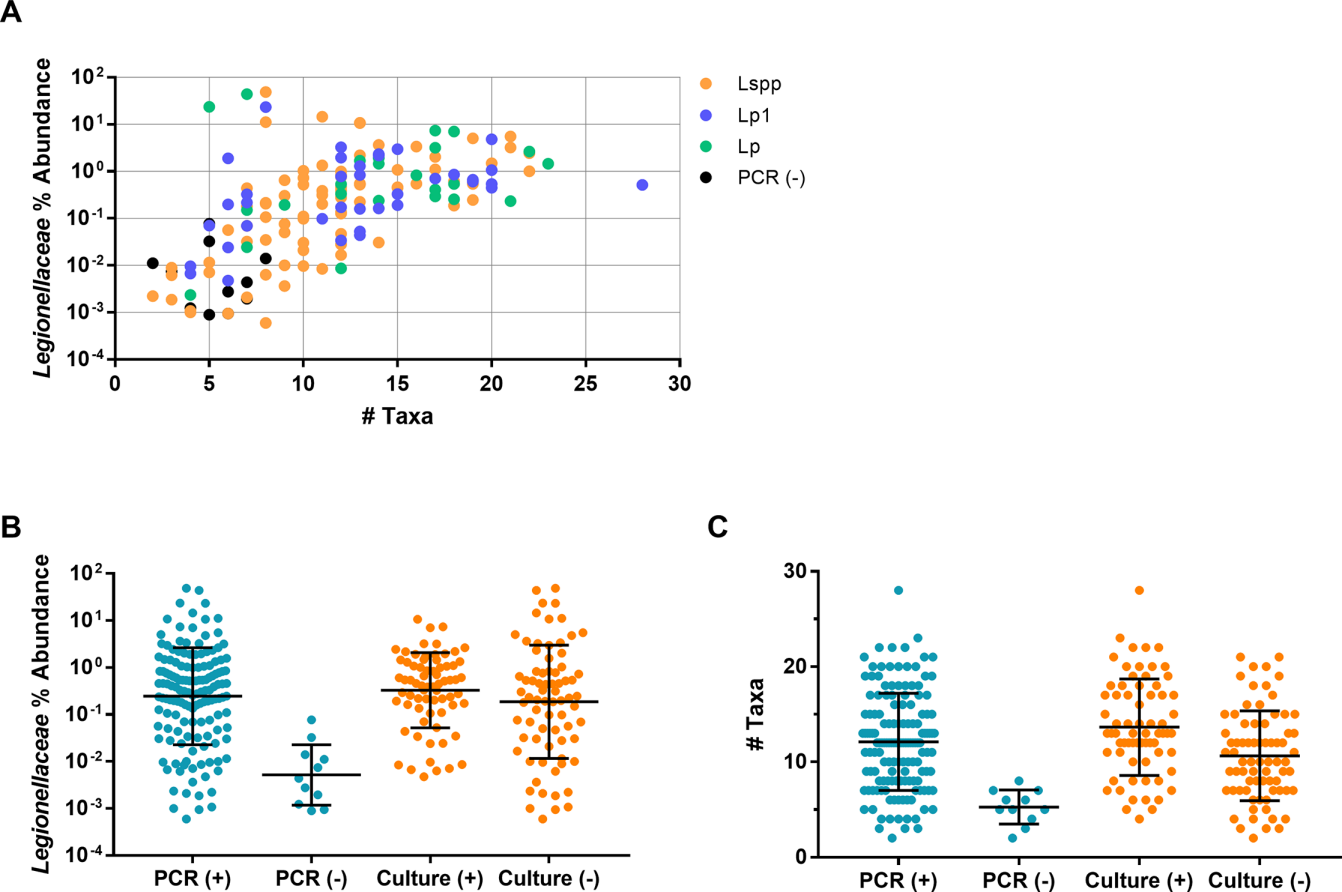
Samples with higher diversity of taxa correlate with higher levels of *Legionellaceae* abundance. PCR positive samples were analyzed by 16S rRNA amplicon sequencing and the relative *Legionellaceae* abundance of each was compared to the number of bacterial families (relative abundance >1%) detected and organized by initial sample multiplex PCR result (A). The relative *Legionellaceae* abundance (B) and number of taxa (C) of samples that were positive or negative for multiplex PCR and culture were analyzed. Bars represent the geometric (B) or arithmetic (C) mean and error bars indicate standard deviation.

16S rRNA amplification and sequencing were successfully performed on 155 CT water samples. We observed a statistically significant positive correlation (r = 0.63, p<0.0001) between the abundance of *Legionellaceae* OTUs and the number of bacterial taxa present in a sample, a trend observed in all PCR-positive samples, regardless of which targets were identified (Fig 3A). The trend of higher relative *Legionellaceae* abundance in samples with more diverse taxa was maintained across all US regions represented in the study (S2A Fig). Comparison of samples by overall PCR result revealed that PCR-negative samples exhibited both low *Legionellaceae* abundances and diversity of other microbial taxa (Fig 3B and 3C). Compared to other taxa, we did not observe a significant difference in the average relative *Legionellaceae* abundances in PCR-positive versus negative samples. However, there was a significant difference in the number of taxa of PCR-positive versus negative samples (p<0.0001), indicating diversity of taxa in a sample positively correlates specifically with the detection of *Legionella* DNA by PCR. Of the subset of samples that were PCR positive, there was no difference in the levels of *Legionellaceae* abundance or taxa diversity between culture-positive and culture-negative samples (Fig 3B and 3C). Additionally, we observed no variation in the *Legionellaceae* relative abundance or diversity of taxa among Lp1, Lp, or Lspp PCR or culture results (data not shown).

### Bacterial phyla present in cooling tower samples are homogenous across US

**Fig 4:**
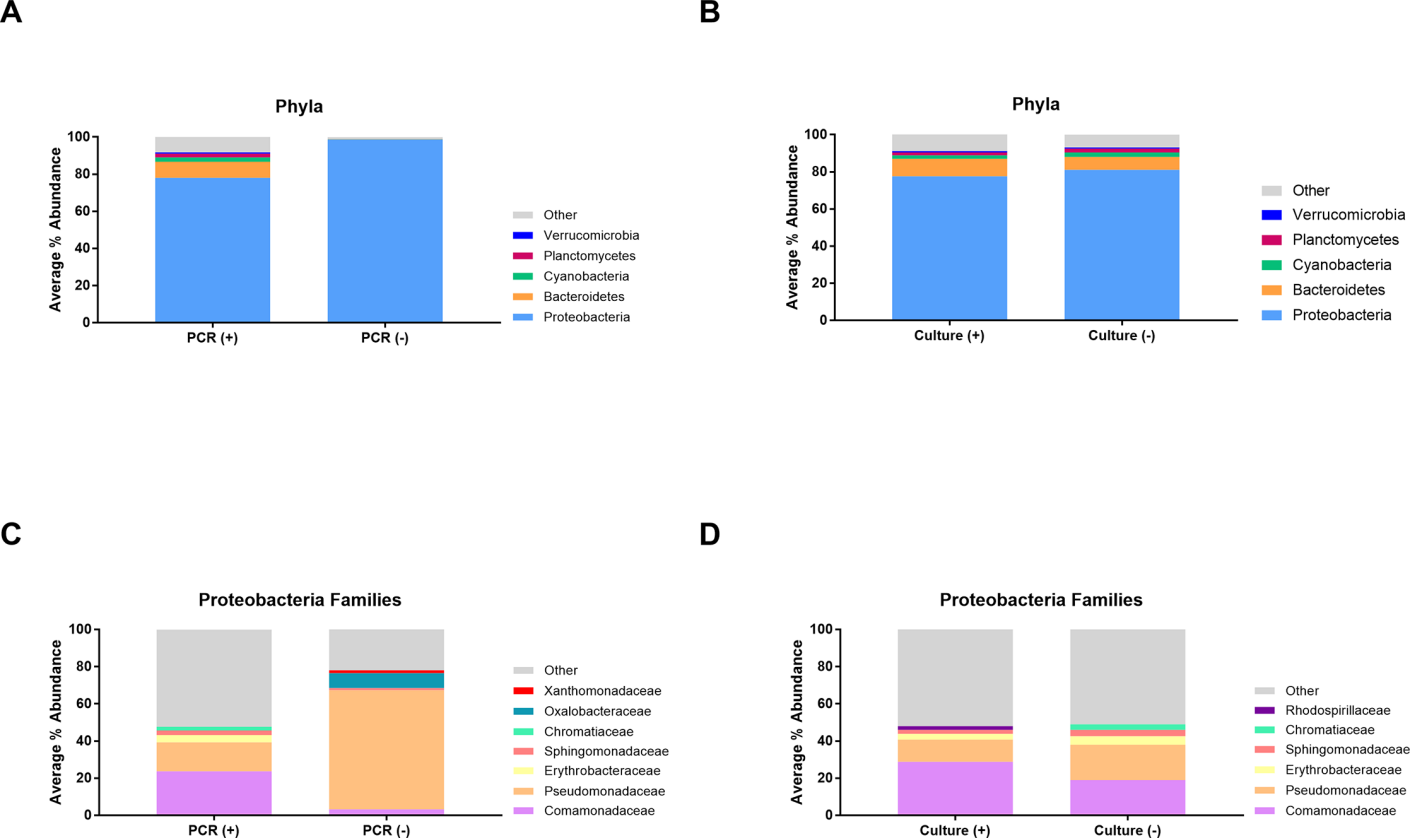
Phyla and Proteobacteria family profiles related to PCR and culture results. The composition of the five most abundant phyla (A, B) and five most abundant Proteobacteria families (C, D) present in cooling tower water samples were compared by overall *Legionella* PCR result (A, C) and culture result (B, D). Note that only samples that were positive by PCR underwent culture.

The top five most abundant bacterial phyla for each sample were identified based on 16S rRNA amplicon sequencing results. The most abundant phyla on average from all samples were: Proteobacteria (79.5%), Bacteroidetes (8.1%), Cyanobacteria (2.2%), Planctomycetes (1.8%), and Verrucomicrobia (0.6%). These abundances were largely consistent across all US climate regions included in the study (S2B Fig). However, the PCR-negative samples had a higher average abundance of Proteobacteria (98.7%) in comparison to the PCR-positive samples (78.0%) (p<0.0001; Fig 4A). Conversely, there were no marked differences between the major phyla of samples that were culture positive versus culture negative (Fig 4B).

DNA from the Proteobacteria phylum represented the majority of DNA abundance in every sample. Notably, this phylum contains *Legionellaceae*. However, analysis of the five most abundant Proteobacteria families per sample revealed the families with highest average abundance were: *Comamonadaceae* (22.2%), *Pseudomonadaceae* (19.0%), *Erythrobacteraceae* (3.7%), *Chromatiaceae* (2.4%), and *Sphingomonadaceae* (2.4%). *Comamonadacaeae* and *Pseudomonadaceae* were among the highest abundance Proteobacteria families across all US climate regions, though there was variation in the other three most abundant families by region (S2C Fig). However, samples in which Lspp were not detected by PCR had a much lower average relative abundance of *Comamonadaceae* (3%) and higher average relative abundance of *Pseudomonadaceae* (64%) compared to the overall *Legionella* PCR-positive samples (24% and 16% for *Comamonadaceae* and *Pseudomonadaceae*, respectively) (Fig 4C). Additionally, 2 of most abundant Proteobacteria families within the PCR-negative samples were different from the overall average profile: *Oxalobacteraceae* (8%), and *Xanthomonadaceae* (1.4%) (Fig 4C). Culture-positive samples had a very similar Proteobacteria family profile to the overall PCR-positive samples while culture-negative samples had a slightly lower abundance of *Comamonadaceae* (19%) and somewhat higher abundance of *Pseudomonadaceae* (19%) than the culture positive samples (29% and 12%, respectively), similar to the trend seen in PCR-negative samples (Fig 4D).

### Microbiome changes over time in a subset of CTs does not correlate with culturability of *Legionella*

**Fig 5:**
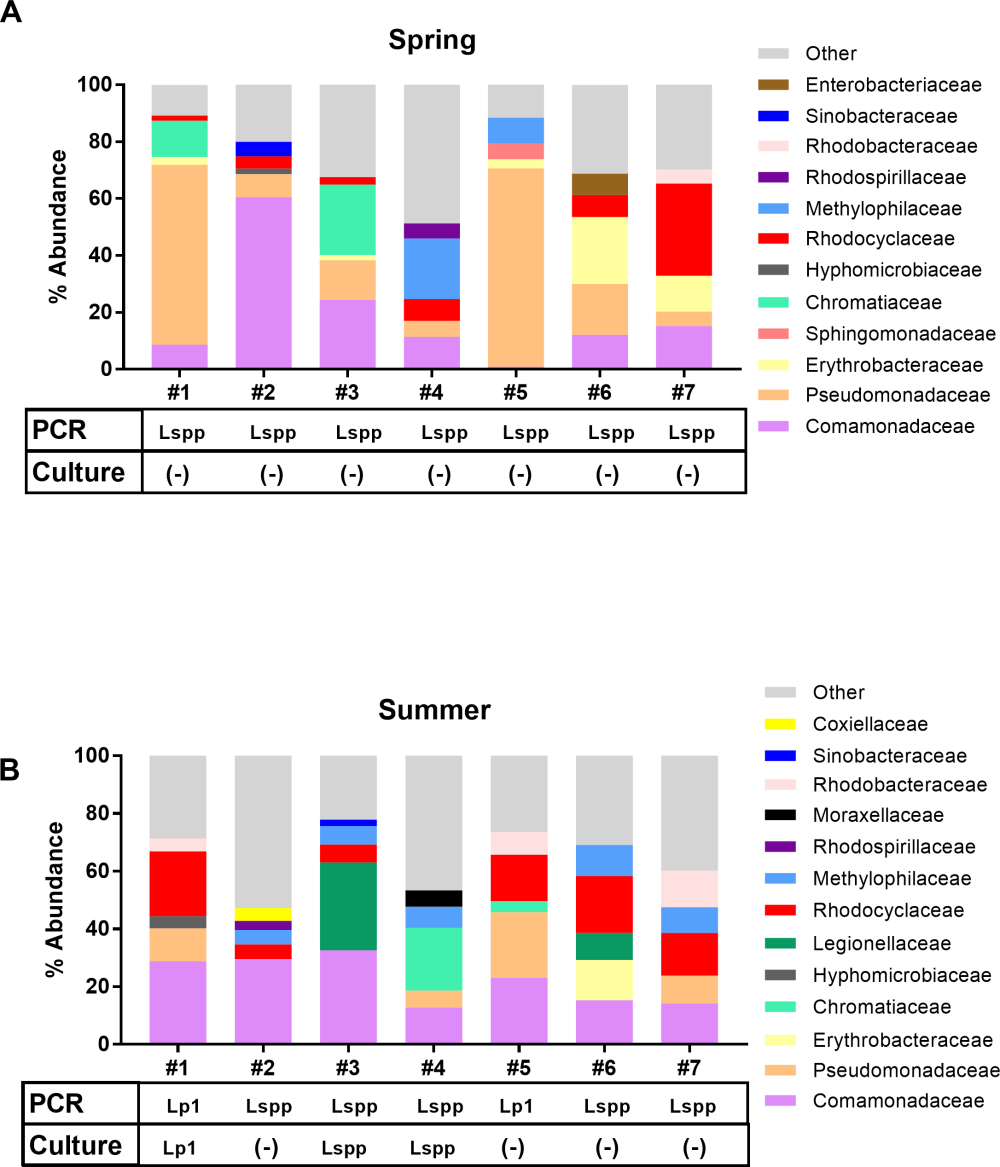
Comparison of Pinellas County CT bacterial family compositions between spring and summer 2016. Multiplex PCR results, culture results, and five highest abundance Proteobacteria family compositions were compared between spring (A) and summer (B) 2016. These samples were PCR-positive for Lspp marker only (Lspp) or all three Lspp, Lp, and Lp1 markers (Lp1). Samples were culture-positive for non-Lp species (Lspp), Lp1 isolates only (Lp1), or were culture-negative [(-)]. In samples where *Legionella* spp. were detected by PCR, *Legionellaceae* was not always among the bacterial families with the highest abundance.

We compared seven CTs from the same region (Pinellas County, Florida, SE) in order to track changes in individual microbial communities over time (Fig 5 and S3 Fig). In April, all of the CTs were PCR-positive for Lspp but no *Legionella* isolates were recovered (Fig 5A). Three months later, two of the CTs became PCR-positive for Lp1, though an Lp1 isolate was only recovered from one of these and Lspp isolates were recovered from two other towers (Fig 5B). However, there was not a clear trend in changes to the composition of the five most abundant bacterial phyla or Proteobacteria families from the CTs that became Lp1 PCR-positive or culture-positive (S3 Fig and Fig 5). Among all seven towers, the most notable change in the phyla profiles between the seasons was an overall increase in the relative abundance of Cyanobacteria and Verrucomicrobia (S3 Fig). Shifts in the highest abundance Proteobacteria families in each tower between seasons were more apparent, with an overall relative abundance reduction of *Pseudomonadaceae* and increase in *Rhodocyclaceae* and *Methylophilaceae* (Fig 5). Two of the towers had a dramatic increase in the relative abundance of *Legionellaceae*, one of which became culture-positive and the other did not (Fig 5). *Legionellaceae* were often not among the five highest abundance Proteobacteria families in these samples and therefore this family is not always included in a sample graph, though *Legionella* was detected by PCR.

## Discussion

CTs are the most frequently reported non-potable water source of LD outbreaks [10] and can involve large numbers of cases [12, 28]. However, the prevalence of *Legionella* in US CTs not associated with disease has not been studied extensively. While our sample set relied on availability from external laboratories, the large number and diverse geographic origins of samples suggest that *Legionella* DNA is common in CT water throughout the US. Assuming that PCR-negative CT samples in this study would also be culture-negative, the overall percentage of culture-positive CT samples examined would be approximately 40% (79/196). A similar proportion of *Legionella* culture-positive CTs has been reported from surveys conducted in Asia, Australia, and Europe [7, 8, 29–31]. Additionally, Lp has also been found to be the most highly recovered *Legionella* species in multiple studies outside of the US [7, 8, 30–32], underscoring the ubiquity of this pathogen within CTs across the globe. Similarity, cooling towers sampled as part of a recent large outbreak investigation in New York City also demonstrated similar detection levels of Lp1 DNA (38%) and isolates (25%) [33].

Multiplex PCR identified over twice as many samples to be positive for *Legionella* DNA than were culture-positive. However, nearly half of the samples that were culture-positive for Lp1 were not identified as Lp1-positive by PCR. This could be due to multiple factors, including possible higher sensitivity of the *Lspp* primers and probe set, quenching of Lp and Lp1 primers in samples with high abundance of non-*pneumophila Legionella* species, or simply an artifact of the automated DNA extraction method used in this study. These findings are important to consider, especially when using PCR to screen waters associated with an Lp1-related LD outbreak response. Our results indicate that, even when screening for Lp1, all samples that are PCR-positive for any *Legionella* marker should be cultured since samples that are only PCR-positive for Lp or Lspp markers may still contain viable Lp1. Future studies may indicate whether or not this difference in detection of Lp1 by PCR and culture is also common among outbreak-related CT waters.

Our findings may have implications surrounding the use of PCR as a screen for routine maintenance of CT water. While maintaining undetectable levels of *Legionella* by culture is often the gold standard for water management, having undetectable levels of *Legionella* DNA may not be a reasonable expectation in all settings. Results from this study show that *Legionella* DNA is present in the majority of CTs sampled from across the US. Consequently, even with an effective water maintenance program, one would expect *Legionella* DNA from killed or inhibited bacteria to be present in CT water. Indeed, out of 196 CT samples, 84% were PCR-positive for *Legionella* but only 47% of these PCR-positive samples were culture positive, suggesting that a substantial portion of the culture-negative CTs contained DNA from *Legionella* that had been rendered un-culturable by standard methods (Fig 2). Whether this indicates loss of viability or a switch to a viable but nonculturable (VBNC) state that still has the potential to cause disease warrants further study. Therefore, presence of *Legionella* DNA alone would not be an accurate indicator of the effectiveness of a water maintenance program. It is tempting to suggest a potential for threshold values for PCR that quantify an abundance of *Legionella* DNA at which remediation would be required. However we did not observe a correlation between the PCR crossing threshold values for detection of *Legionella* DNA and the ability to culture *Legionella* bacteria. It should be noted that this study was limited by variation in sample collection methods and differences in time between collection and CDC processing, which could affect DNA integrity and viability of *Legionella*. In addition, we did not perform amoebic co-culture to increase recovery of VBNC bacteria. Future studies with rigorous collection and testing timeframes and more aggressive recovery techniques may provide further insight into these issues.

The results of this study were also limited by the possibility of selection bias in the sample set we received from CTs undergoing routine water testing. One may suspect that CTs undergoing routine testing are likely to be covered by a water management program and therefore might be better managed and have less detectable *Legionella* than CTs that are not routinely screened. Conversely, whether or not some of the included CT samples were collected because of previous concerns about *Legionella* is unknown. Additionally, PCR-negative samples were not cultured in this study and it is possible that some of these samples may have also yielded *Legionella* isolates.

In this study, Lp1 was overall the most commonly isolated serogroup of the most frequently cultured *Legionella* species. Of note, 72% of samples where Lp1 was recovered also contained at least one other Lp or Lspp, indicating a possible correlation between diversity of Lspp in water and culturability of Lp1. This percentage may be an underestimate as it has been reported that the growth conditions used in this study (BCYE agar, 2.5% CO_2_, 35°C) are not optimal for some non-*pneumophila* species [34]. Therefore, if a non-Lp *Legionella* spp is isolated from a CT, the risk of concurrent or future growth of Lp1 should be considered high and water management procedures reviewed. Further research is needed to assess the relationship of non-Lp *Legionella* spp on Lp presence in environmental sources. Notably, van der Mee-Marquet *et al*. [35] reported that the presence of *L*. *anisa* potentially hindered the ability to isolate Lp and therefore the detection of non-*pneumophila* species may indicate a risk for Lp co-contamination. Additionally, all of the Lspp that have been identified in the literature are considered to be potentially pathogenic [5], especially for certain high-risk groups such as immunocompromised persons. Careful maintenance of CTs with water management programs designed to monitor water quality parameters and ensure adequate disinfection is recommend to reduce risk (https://www.cdc.gov/legionella/maintenance/wmp-toolkit.html).

Microbiome analyses using 16S amplicon sequencing showed an overall positive correlation between the relative abundance of *Legionella* in CT microbiomes and the diversity of the taxa present. In addition, results indicated that diversity of taxa in a sample positively correlates specifically with the detection of *Legionella* DNA by PCR. This correlation is not surprising given that *Legionella* is a fastidious organism and the environment in which it thrives (warm water with high concentration of nutrients or sediment, established biofilm, and amoebae for replication [1]) would likely be permissive to many other bacterial taxa. Alternatively, low biodiversity samples could be dominated by a few microorganisms that outcompete or exclude *Legionella* spp.

Additionally, some differences were observed in the bacterial community composition present in PCR-positive versus PCR-negative samples. Overall, PCR-negative samples contained a higher average abundance of Proteobacteria than the PCR-positive samples. Furthermore, within the Proteobacteria phylum, PCR-negative samples had a much higher average abundance of *Pseudomonadaceae* and lower abundance of *Comamonadaceae*. It is possible that the presence of *Pseudomonadaceae* members, commonly found in biofilm, have an antagonist effect on *Legionella* populations. Notably, these results are limited by the low number of PCR-negative samples (n = 32) available for analysis. Further studies that include a higher number of PCR-negative samples will help refine these observations.

Unexpectedly, the composition of bacterial phyla and Proteobacteria families within the microbiome of CTs proved to be remarkably homogenous across all US climate regions included in the study. This finding indicates that climate and geography may have little influence on the communities present in CTs. Interestingly, a microbiome survey of industrial CTs from Queensland, Australia also reported Proteobacteria and Bacteroidetes to be among the most abundant phyla, though Firmicutes were the overall dominant taxa [17]. This suggests that there may be broad similarities in CT microbial communities not only in the US but across the globe. Additionally, we did not see an obvious trend in the microbiomes of three individual CTs (sampled in different seasons) that shifted from culture-negative to culture-positive over time, a finding supported by the results of Wéry et al. [16] that the microbial community structure of a CT was unchanged during Lp proliferation. However, our findings involved a small number of CTs examined over time and further studies are needed to provide robust support for these initial results.

We have shown that *Legionella* bacteria DNA is common in a large sample of US CTs not known to be associated with disease, and Lp1 could be cultured from nearly 1 in 4 of the CTs with *Legionella* DNA detected. This suggests that, on its own, the presence of *Legionella* in a CT is not sufficient to cause disease and other dynamics, such as cooling tower design and proximity of a susceptible population, may contribute to a CT with *Legionella* becoming the source of LD outbreak. Additionally, our findings suggest that the high reported LD incidence in some parts of the country and low reported incidence in others [36] is likely not due to differences in the distribution of *Legionella* in CT waters. The geographic disparity in reported LD incidence is more likely due to several factors, including regional differences in use of cooling towers, *Legionella* exposure from other aspects of the built environment, population density and susceptibility, frequency in LD diagnostic testing, and public health surveillance and reporting. However, it is important to note that in some regions our ability to examine *Legionella* detection in CTs was limited by a small number of samples.

Altogether, our findings underscore the high frequency of *Legionella* in CTs throughout the US and the homogeneity of the microbiomes in these CTs. Therefore, the potential exists for LD cases and outbreaks to occur across the continental US wherever a colonized CT and a susceptible population coincide. However, CT waters specifically associated with outbreaks may have differences in *Legionella* abundances and types and may contain altered microbial communities compared to our findings. More focused research on CTs with different designs and in various locations over time will likely further contribute to our understanding of these dynamic systems. Detailed examination of the microbiome and physical parameters such as water quality metrics may help elucidate what factors cause *Legionella* in a CT to shift from environmental bacteria to outbreak pathogens.

## Supporting information

S1 Fig Sample PCR crossing threshold values by culture result. Multiplex PCR crossing threshold values for the 23S rRNA pan-*Legionella* marker were compared between culture-positive [Culture (+)] and culture-negative [Culture (-)] samples. NS = not significant (Mann-Whitney test, p = 0.54).
(TIF)

S2 Fig 16S rRNA amplicon sequencing results by US climate region. Samples were analyzed by 16S rRNA amplicon sequencing and the relative *Legionellaceae* abundance of each was compared to the number of bacterial families (relative abundance >1%) detected and organized by US climate region (A). The five highest abundance phyla (B) and five highest abundance families from the Proteobacteria phylum (C) were identified for every sample in each region.
(TIF)

S3 Fig Comparison of Pinellas County CTs bacterial phyla compositions between spring and summer 2016. Multiplex PCR, culture, and the five highest abundance phyla compositions were compared between spring (A) and summer (B) 2015. TM7 is a candidate phylum.
(TIF)
