# Supplementary figures and images for: Distribution of Legionella and bacterial community composition among regionally diverse US cooling towers
Source: PLoS One. 2017 Dec 20;12(12):e0189937. doi: 10.1371/journal.pone.0189937 (PMC5738086; doi:10.1371/journal.pone.0189937)

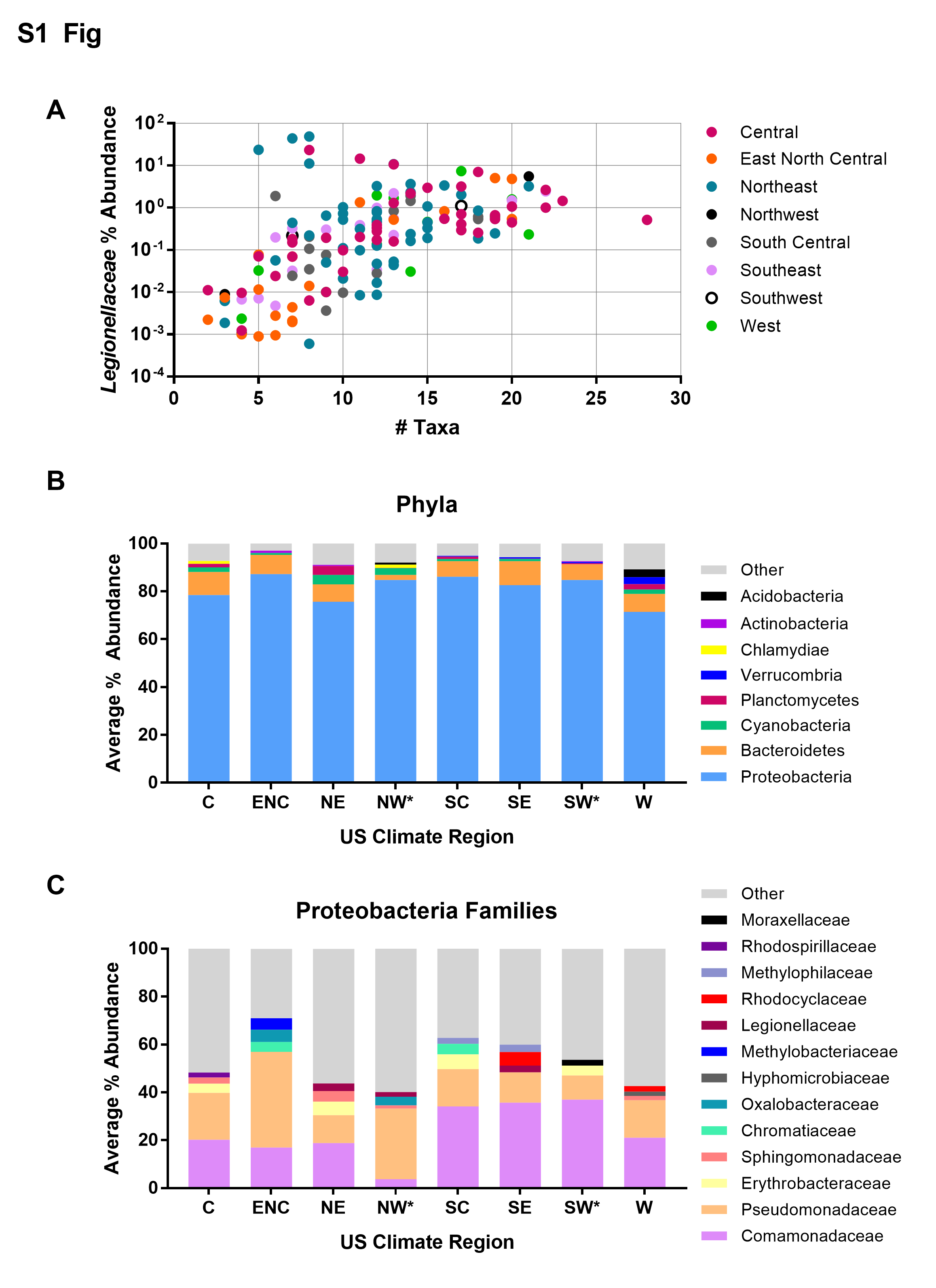

Supplement: S1 Fig — Multiplex PCR crossing threshold values for the 23S rRNA pan-Legionella marker were compared between culture-positive [Culture (+)] and culture-negative [Culture (-)] samples. NS = not significant (Mann-Whitney test, p = 0.54). (TIF) [file pone.0189937.s001.tif]

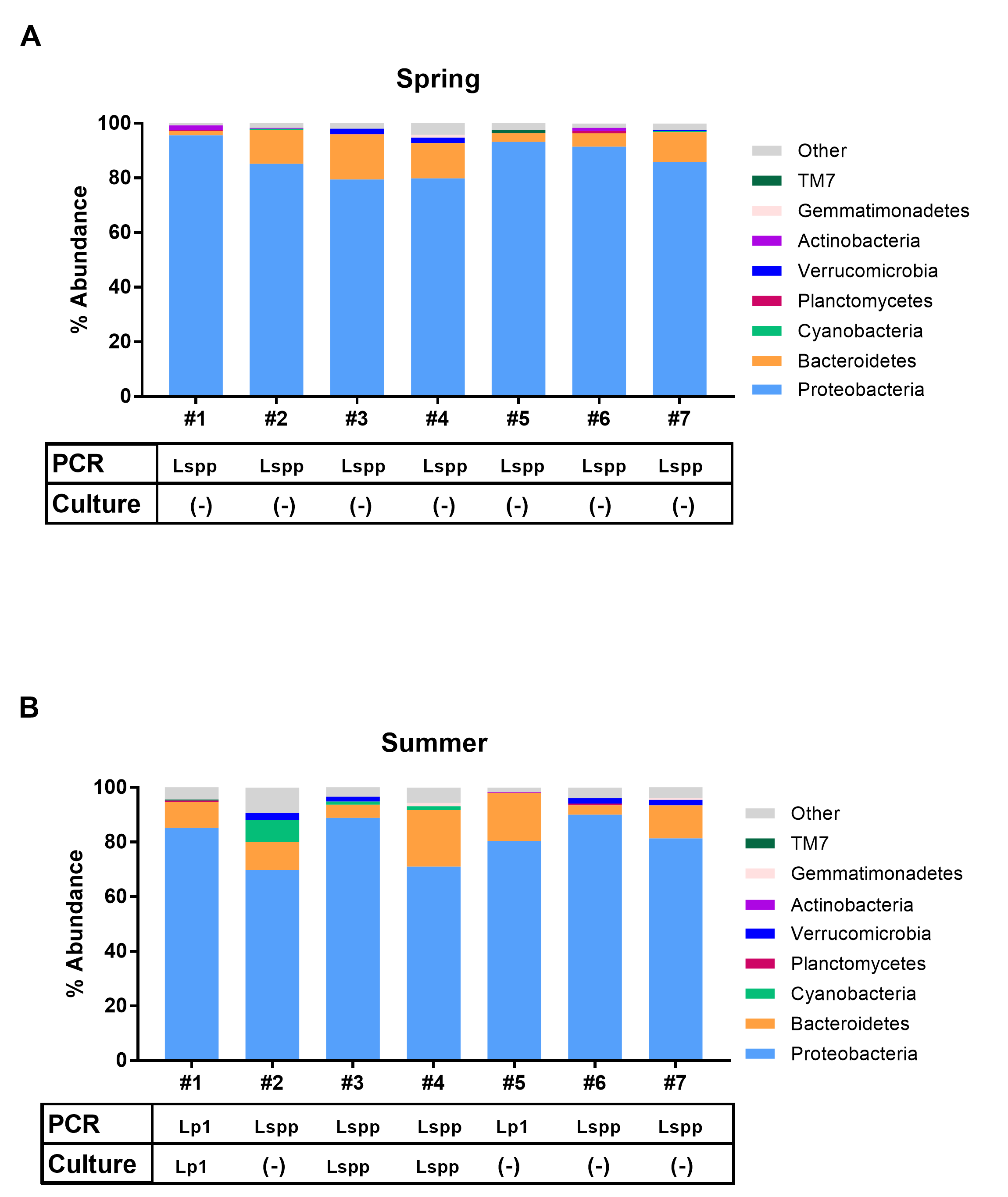

Supplement: S2 Fig — Samples were analyzed by 16S rRNA amplicon sequencing and the relative Legionellaceae abundance of each was compared to the number of bacterial families (relative abundance >1%) detected and organized by US climate region (A). The five highest abundance phyla (B) and five highest abundance families from the Proteobacteria phylum (C) were identified for every sample in each region. (TIF) [file pone.0189937.s002.tif]

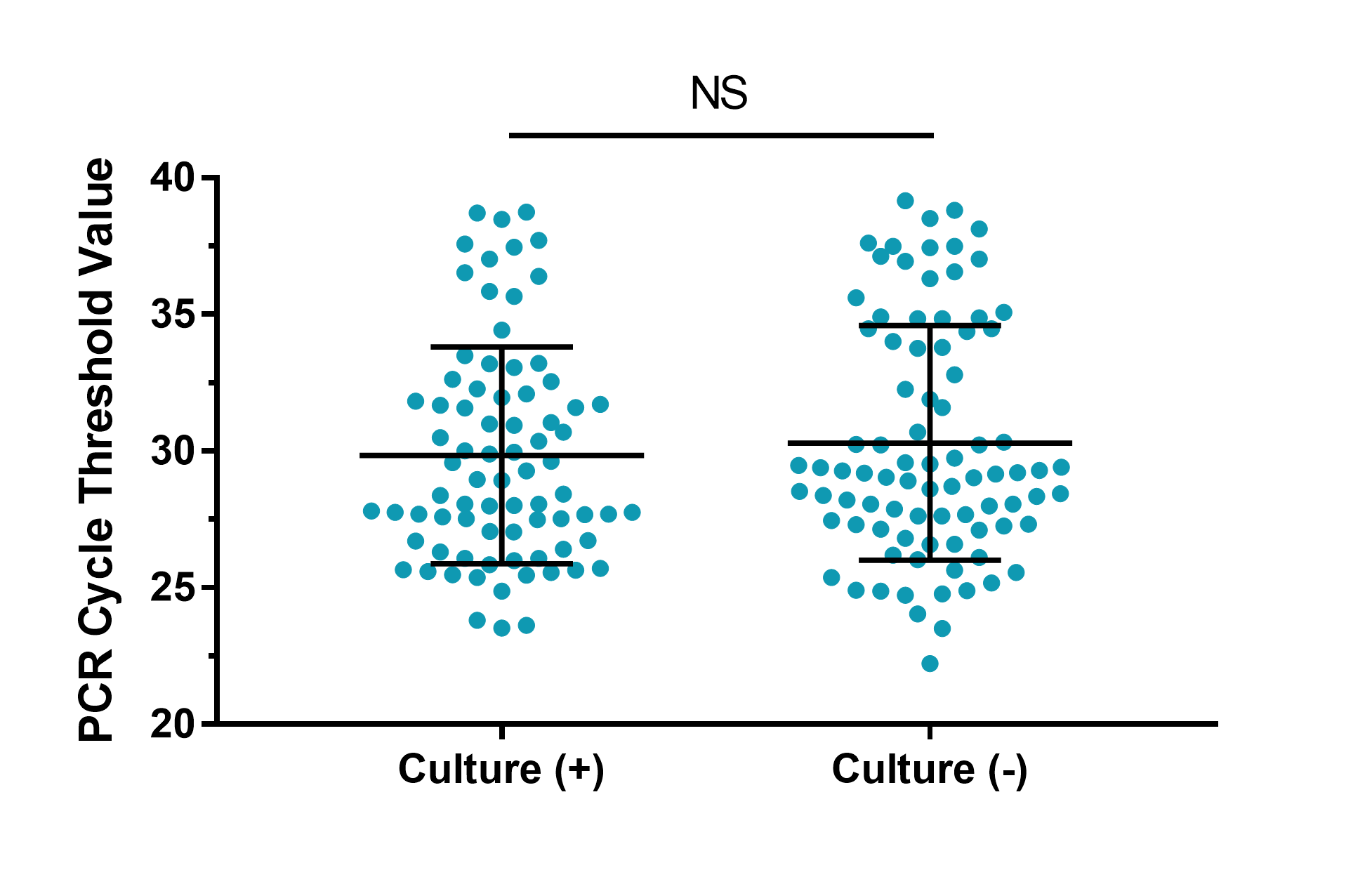

Supplement: S3 Fig — Multiplex PCR, culture, and the five highest abundance phyla compositions were compared between spring (A) and summer (B) 2015. TM7 is a candidate phylum. (TIF) [file pone.0189937.s003.tif]
